# Efficient Isolation of Cellulose Nanocrystals from Seaweed Waste via a Radiation Process and Their Conversion to Porous Nanocarbon for Energy Storage System

**DOI:** 10.3390/molecules29204844

**Published:** 2024-10-13

**Authors:** Jin-Ju Jeong, Jae-Hun Kim, Jung-Soo Lee

**Affiliations:** Department of Bio-Chemical Engineering, Chosun University, Chosundaegil 146, Dong-gu, Gwangju 61452, Republic of Korea

**Keywords:** cellulose nanocrystal, seaweed, electron beam irradiation, porous carbon, electrical double layer capacitor

## Abstract

This article presents an efficient method for isolating cellulose nanocrystals (CNcs) from seaweed waste using a combination of electron beam (E-beam) irradiation and acid hydrolysis. This approach not only reduces the chemical consumption and processing time, but also improves the crystallinity and yield of the CNcs. The isolated CNcs were then thermally annealed at 800 and 1000 °C to produce porous nanocarbon materials, which were characterized using scanning electron microscopy, X-ray diffraction, Raman spectroscopy, and X-ray photoelectron spectroscopy to assess their structural and chemical properties. Electrochemical testing of electrical double-layer capacitors demonstrated that nanocarbon materials derived from seaweed waste-derived CNcs annealed at 1000 exhibited superior capacitance and stability. This performance is attributed to the formation of a highly ordered graphitic structure with a mesoporous architecture, which facilitates efficient ion transport and enhanced electrolyte accessibility. These findings underscore the potential of seaweed waste-derived nanocarbon as a sustainable and high-performance material for energy storage applications, offering a promising alternative to conventional carbon sources.

## 1. Introduction

Global industrialization and the overuse of non-renewable energy have led to a significant release of greenhouse gasses, increasing global temperatures and contributing to widespread environmental degradation. Furthermore, global greenhouse gas emissions, particularly CO_2_ from fossil fuels, are projected to increase by 50% by 2050. Without technologies or strategies to mitigate CO_2_ emissions, atmospheric concentrations and global temperatures, both surface and ocean, will continue to rise. The warming caused by greenhouse gasses has already led to severe impacts, including species extinction, biodiversity loss, droughts, floods, wildfires, ocean acidification, glacier melting at the poles, and rising sea levels. To address the escalating levels of greenhouse gasses and the accompanying rise in global temperatures, 197 countries reached a unanimous agreement at the Paris Climate Change Conference (PCCC) in 2015. This agreement, known as the Paris Agreement, outlines a global strategy for climate action beyond 2020. To achieve the goals set by the Paris Agreement and promote sustainable development, it is important not only to reduce CO_2_ emissions but also to extract CO_2_ from the atmosphere. To attain net-zero or negative carbon emissions, various strategies must be implemented across social, economic, environmental, and technological fields. Among these strategies, technological and industrial approaches are emphasized as primary methods to be adopted, such as reducing energy consumption, utilizing alternative energy sources, decreasing reliance on fossil carbon sources, and recycling and reusing waste. Reducing energy consumption and dependency on fossil carbon sources often necessitates significant capital investment, the installation of additional infrastructure, and the resolution of various logistical challenges. In contrast, waste recycling presents a more accessible and immediately implementable approach to reducing carbon emissions [1,2,3].

Among approaches, biomass recycling plays a key role in achieving carbon neutrality through several mechanisms, such as its involvement in the carbon cycle, its potential to replace fossil fuels, and its ability to facilitate carbon capture and storage. Further, biomass recycling also aids in waste management, resource recycling, and the preservation and restoration of ecosystems. These attributes contribute to the development of sustainable energy systems while promoting environmental protection, allowing both goals to be pursued in parallel [4,5].

As of recent, cellulose nanocrystals (CNcs) are exceptionally promising materials that exhibit superior transparency, biodegradability, surface area, and strength properties compared with those of traditional cellulose [6,7,8,9]. CNcs are predominantly isolated from wood biomass waste. The isolation of CNcs from wood biomass waste requires rigorous pre-treatment involving a strong base, high pressure, heat, and considerable energy to remove other constituents, such as hemicellulose, lignin, and other ingredients. This intensive process also yields a disappointingly low amount of CNcs. In contrast, the CNcs obtained from seaweed biomass are naturally low in lignin, hemicellulose, and other residual substances, which results in a more efficient and environmentally friendly extraction process [10,11,12,13].

Seaweed biomass provides a lot of benefits for sustainable progress and environmental conservation [14]. It is a rapidly renewable resource that can be harvested extensively without causing deforestation. Moreover, seaweed plays a significant role in absorbing atmospheric carbon dioxide, helping to mitigate greenhouse gas levels. However, seaweed harvested during summer becomes inedible due to an increased cellulose content, owing to rising sea temperatures. These inedible and harmful seaweeds pose significant ecological and economic challenges. By inhibiting the growth of other marine organisms, such as coral reefs, they disrupt the marine ecosystem and reduce biodiversity. The fishing industry faces complications due to net seaweed entanglement and blocked navigation routes, resulting in decreased catch volumes and financial losses. Tourism also suffers when seaweed accumulates on beaches, emitting unpleasant odors, which diminishes their appeal. As seaweed decomposes, it depletes oxygen and alters the chemical balance of seawater, further stressing the marine life. Aquaculture is similarly impacted, with the health and growth of farmed species being compromised, leading to additional economic setbacks. The removal and disposal of this inedible seaweed impose a further financial burden on local economies. To resolve these problems, it is important to effectively recycle harmful seaweed waste and convert it into high-value materials. Addressing these challenges requires ongoing monitoring, effective management, targeted policies, and dedicated research efforts [15,16,17,18].

Generally, the isolation of CNcs from wood biomass can be achieved through both chemical and mechanical techniques. In mechanical processing, the homogenizer operated at a high pressure and speed can disrupt the hydrogen bonds between the crystalline regions of cellulose. On the other hand, ball milling uses the physical force of high-energy balls to defibrillate cellulose. However, this method is energy intensive and produces CNcs with a limited size distribution, low thermal properties, and low crystallinity and strength. CNcs are often mass-produced using a strong acid and chemical catalyst to hydrolyze cellulose. These chemical agents particularly oxidize the primary -OH group to a -COO group, while the -OH groups of cellulose form a -O-SO_3_^−^ with sulfuric acid, facilitating the isolation of CNcs. Despite their effectiveness, chemical methods have significant disadvantages, including high costs, lengthy reaction times, and environmental pollution resulting from substantial chemical usage [19,20,21]. Consequently, researchers are in pursuit of ecofriendly, simple, high-production, and economically effective methods for CNc production such as plasma, gamma radiation, electron beam irradiation, etc. [22,23,24].

Among these, electron beam (E-beam) irradiation is a novel technique in which ions are produced in a material to cause a variety of chemical reactions, achieving chemical bond cleavage [25,26]. This ionizing radiation causes the scission of molecular chains, degradation into smaller components, and depolymerization of polymer chains into monomers or shorter polymers of cellulose predominantly. The degree of this degradation is proportional to the radiation dose. Extensive research has been conducted to understand the relationship between the radiation dose and cellulose degradation. These studies have consistently revealed that higher radiation doses lead to more intense cellulose degradation. This phenomenon is attributed to the higher energy input, which enhances the ionization and subsequent chemical reactions that dismantle the chemical structure of cellulose. Such findings are crucial for optimizing the use of radiation for cellulose modification in industrial settings or for the treatment of cellulose-based biomass waste [27,28,29,30].

Recently, CNcs and their derivatives have become promising candidates for components in energy-related devices. Due to their distinct mechanical and electrochemical characteristics, CNcs are well-suited for various components, such as electrolytes, separators, binders, and substrates. Moreover, through carbonization, CNc-derived carbon can be converted into highly conductive materials, which have been effectively employed as electrodes or current collectors in sustainable energy storage systems.

In this study, we investigated a facile method for isolating CNcs from seaweed waste, whereby different particle sizes were obtained depending on the reaction time and molarity. We found a new and highly effective approach of E-beam irradiation for isolating CNcs from seaweed waste, with a significant decrease in the chemical reagent usage and processing time. Then, a porous nanocarbon material was obtained by annealing the prepared CNcs, and it was applied as an electrode material in an electrical double-layer capacitor (EDLC). This study provides guidelines for the effective isolation of CNcs from seaweed biomass and highlights its potential as a next-generation core material for other industrial and research fields.

## 2. Results and Discussion

**Figure 1:**
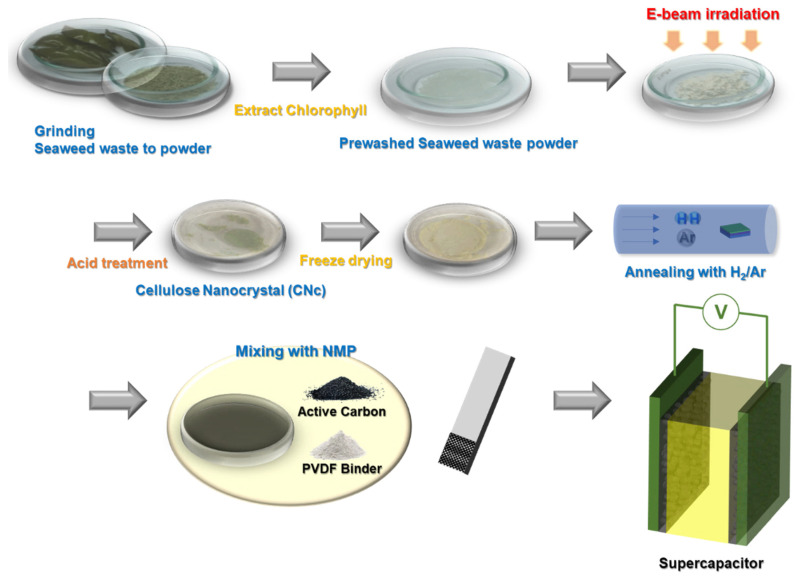
Schematic illustration of the isolation process of the CNcs from inedible seaweed waste and its application.

Figure 1 illustrates the process of isolating CNcs from seaweed waste and its subsequent electrochemical application. The seaweed waste was thoroughly washed with deionized water and then fully dried in a vacuum oven. Once dried, the seaweed waste was finely ground to improve the efficiency of the subsequent chemical reactions. The powdered seaweed waste was stirred in EtOH at 80 °C for 1.5 h to remove chlorophyll [31,32,33]. Owing to its aromatic structure, chlorophyll exhibits resistance to E-beam irradiation, which diminishes the effectiveness of the E-beam in inducing structural modification or degradation. The stability provided by the delocalized π-electron system in the aromatic rings of chlorophyll contributes to its reduced reactivity under E-beam exposure, limiting the extent of its breakdown during irradiation [34,35]. After the seaweed waste was washed, E-beam irradiation was performed to improve the crystallinity of the CNcs and reduce the environmental impact and processing time. This method is advantageous, owing to its speed and convenience, and eliminates the need for chemical additives to achieve the desired modifications. As the E-beam dose increased, the size of the CNcs decreased significantly. The dose, representing the energy absorbed per unit mass, is measured in kilograys (kGy), with one kGy being equivalent to the absorption of 1 J/g of the material. For instance, when cellulose is irradiated to 1 kGy dose of E-beam, each g absorbs 1 J from the radiation. Thus, E-beam irradiation stimulated the isolation of CNcs with enhanced properties and an improved yield, without the demand for excessive chemical reagents. Subsequently, the E-beam-irradiated seaweed waste was hydrolyzed with an acidic solution, aqueous H_2_SO_4_ with a small quantity of H_2_O_2_ at 45 °C, for different durations.

The seaweed waste subjected to the two-step treatment, that is, processed by E-beam irradiation and acid hydrolysis, was then centrifuged and washed with distilled water to neutralize the solution and remove the unreacted material. The resulting slurry was dispersed in tert-butanol, and the CNc powder was finally obtained by freeze-drying. The CNc powder was then thermally annealed in a mixed Ar/H_2_ gas atmosphere at 800 or 1000 °C for 30 min under isothermal conditions, which facilitated the formation of high-quality porous carbon. After high-temperature annealing, the carbonized CNc powder was treated with a mild etchant to produce a porous nanocarbon material. This method of producing highly crystalline porous carbon structures from seaweed waste is simple and efficient, which makes it highly suitable for industrial-scale production.

**Figure 2:**
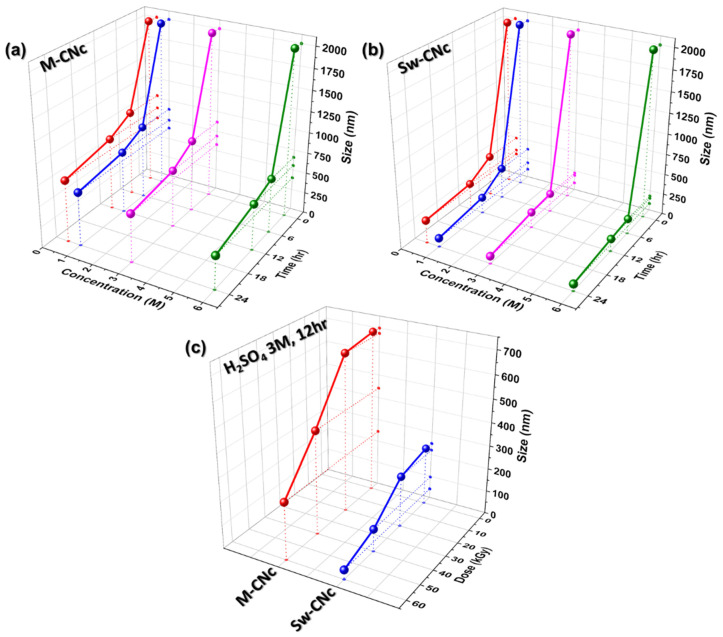
CNc size as a function of the reaction time, acid molarity, and E-beam dose, as determined by DLS. (**a**) M-CNcs and (**b**) Sw-CNcs based on the reaction time and acid molarity; (**c**) E-beam-irradiated M-CNcs and Sw-CNcs under optimal conditions for acid hydrolysis.

Dynamic light scattering (DLS) is a valuable technique for measuring the size distribution of CNc. Figure 2 presents the CNc particle sizes as functions of the reaction time, acid molarity, and E-beam dose. With increasing reaction time and acid molarity, the CNc particle size initially decreased, indicating that longer reactions and higher acid molarity facilitate the breakdown of cellulose into smaller CNc particles. However, the size decreased after 12 h of hydrolysis at 3 M acid concentration, as shown in Figure 2a,b, highlighting the optimal conditions for minimizing the particle size of CNcs and avoiding overreaction. However, compared with that of the M-CNcs, the particle size of the Sw-CNcs decreased more significantly at 12 h under 3 M acid conditions. This result suggests that the smaller size of the Sw-CNcs under optimal conditions was due to their superior wettability and the more flexible tissue of the seaweed, which accelerated the hydrolysis reaction. The optimal reaction time and acid concentration were determined to be 12 h and 3 M, respectively.

A further decrease in the size of the CNc particles was achieved through the prior E-beam irradiation of the MC and seaweed waste at 20, 40, and 60 kGy. After E-beam irradiation, each sample was subjected to acid hydrolysis under the optimal hydrolysis conditions. As the E-beam irradiation dose increased, the particle sizes of both Sw-CNcs and M-CNcs decreased significantly. Ultimately, CNcs with a size of approximately 100 nm were successfully isolated from the seaweed waste using a combination of E-beam irradiation and acid hydrolysis, as shown in Figure 2c.

**Figure 3:**
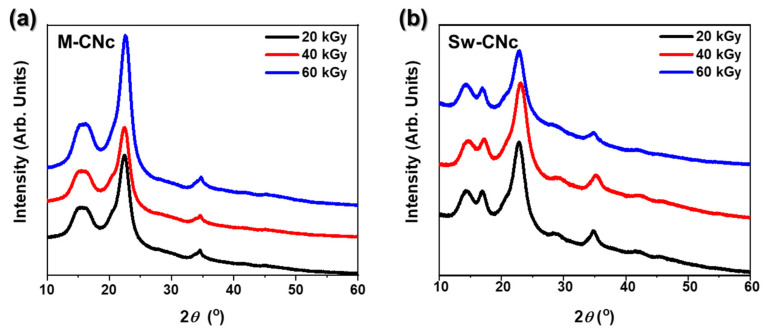
XRD patterns of (**a**) M-CNcs and (**b**) Sw-CNcs according to the E-beam irradiation dose.

Crystallinity is an important parameter that determines the physical properties of materials, such as their strength and stability. Figure 3 shows the XRD patterns of M-CNcs and Sw-CNcs after acid hydrolysis under optimal conditions, highlighting how their crystallinity changed when irradiated with different E-beam doses. The Sw-CNc samples consistently displayed a strong double peak between 15° and 20°, indicating a well-ordered crystalline structure. In contrast, M-CNcs showed a single peak in this range, suggesting lower crystallinity and the presence of non-crystalline regions. At 23.6°, the peak for M-CNcs became sharper and more pronounced as the E-beam dose was increased from 20 to 60 kGy, indicating that the non-crystalline regions were effectively degraded, while the crystalline structure remained intact. This sharper peak is related to the larger particle size of M-CNcs and indicates its well-defined crystalline regions [36,37]. However, when irradiated at 60 kGy, the overall peak intensity of Sw-CNcs decreased significantly, indicating that high-dose E-beam irradiation caused the crystalline structure to collapse. This suggests that excessive irradiation can damage the sample. Therefore, an E-beam dose of 40 kGy was determined to be optimal for isolating CNcs while maintaining its crystalline structure without significant degradation. This balance allows for the effective breakdown of the non-crystalline regions while preserving the overall structure of the sample.

**Figure 4:**
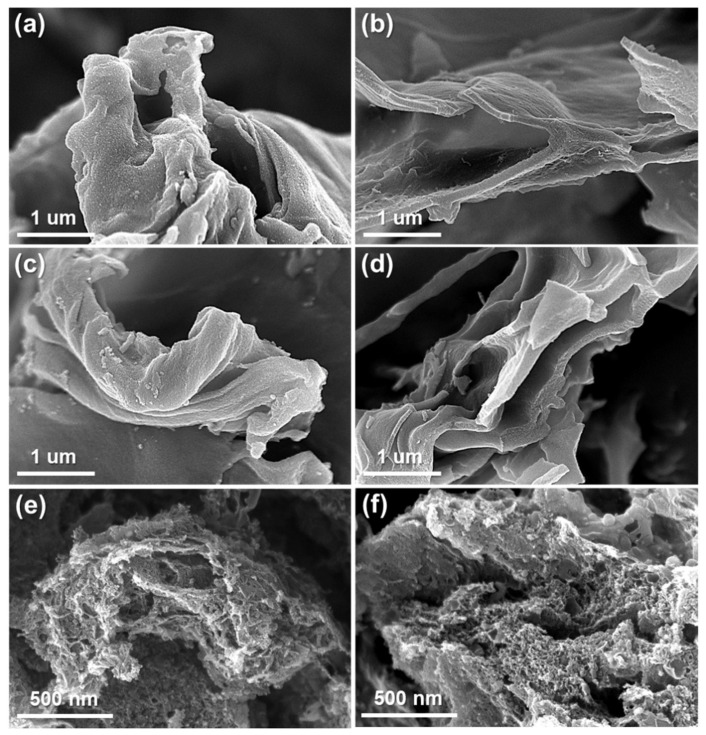
Morphologies of the porous nanocarbon materials obtained by varying the CNc type and annealing temperature. SEM images of (**a**) M-CNc 800, (**b**) Sw-CNc 800, (**c**) M-CNc 1000, and (**d**) Sw-CNc 1000; magnified images of (**e**) M-CNc 1000 and (**f**) Sw-CNc 1000.

Figure 4 presents the morphological characteristics of the porous nanocarbon materials obtained by annealing the different CNcs at 800 and 1000 °C. Figure 4a shows that M-CNc 800 exhibits considerable agglomeration, primarily because of its larger particle size, which decreases the available surface area and promotes particle clustering. In contrast, Figure 4c shows that M-CNc 1000 underwent significantly less agglomeration. This is likely due to the increased degree of carbonization at 1000 °C, which promotes a more ordered carbon structure and enhances particle stability, thereby reducing the propensity for aggregation. Similar trends were observed for Sw-CNc 800 and Sw-CNc 1000, as shown in Figure 4b,d. However, the Sw-CNc-based samples exhibited smaller particle sizes than the M-CNc-based ones, exhibiting a higher surface area and more dispersed particle morphology.

The reduced particle size of the Sw-CNcs effectively minimizes particle agglomeration, even at lower annealing temperatures. In the magnified images, the Sw-CNcs 1000 (Figure 4f) demonstrates finer and more uniformly distributed pores than M-CNc 1000 (Figure 4e). The uniform distribution and smaller pore size of Sw-CNc 1000 are advantageous for improving its electrochemical performance. The superior pore architecture of Sw-CNc 1000 ensures better accessibility of the active surface area by the electrolyte, thereby enhancing the overall energy-storage capabilities and improving the rate performance of the material.

**Figure 5:**
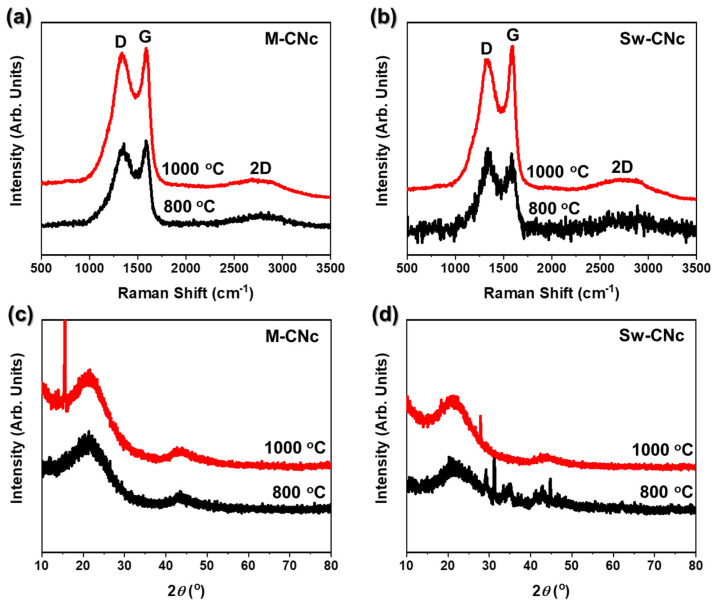
Characterization of the porous nanocarbon materials obtained from CNc. Raman spectra and XRD patterns, respectively, of the carbon materials derived from (**a**,**c**) M-CNcs and (**b**,**d**) Sw-CNcs using different annealing temperatures.

Raman spectroscopy is a representative non-destructive analytical method that is primarily used to measure the quality of carbon. The Raman spectra presented in Figure 5 allow for an in-depth analysis of the structural properties and quality of the porous nanocarbon materials. Two key peaks were identified: the D band centered at approximately 1350 cm^−1^ and the G band located near 1580 cm^−1^. The D band is associated with first-order zone-boundary phonons and arose from structural disorder in the sp^2^-hybridized carbon framework. This disorder is typically attributed to defects in the carbon lattice, such as vacancies, edge sites, and distortions in the sp^2^ C–C bonding network. The intensity of the D band reflects the degree of disorder and imperfections in the material. On the other hand, the G band corresponds to the E2g phonon mode of sp^2^ carbon atoms and is indicative of the in-plane vibrations of sp^2^-hybridized C–C bonds. This band represents the graphitic domain, in which the carbon atoms are arranged in a highly ordered planar structure [38,39].

The intensity and sharpness of the G band are directly related to the degree of graphitization and crystalline order within the carbon matrix. The intensity ratio of the D to G band (I_D_/I_G_) is a commonly used parameter to evaluate disorder in carbon-based materials [40]. A higher I_D_/I_G_ ratio indicates a greater concentration of defects, whereas a lower ratio indicates a more crystalline, well-ordered carbon structure. As shown in Figure 5a,b, the overall intensity of the two bands increased, and the peaks became narrower with increasing annealing temperature. This behavior suggests that the structural quality of the porous nanocarbon material improved as the carbonization temperature increased. Specifically, the D and G bands of M-CNc 1000 (Figure 5a) exhibit comparable intensities, leading to a relatively high I_D_/I_G_ ratio, which indicates a higher degree of disorder within the carbon structure. In contrast, the D and G bands of Sw-CNc 1000 (Figure 5b) demonstrate a lower I_D_/I_G_ ratio, owing to the significantly higher intensity of the G band relative to that of the D band. The lower I_D_/I_G_ ratio of Sw-CNc 1000 reflects the reduced structural defects and a higher degree of graphitic ordering. Furthermore, the G band of Sw-CNc 1000 is narrower and more defined than that of M-CNc 1000, suggesting superior carbon crystallinity. This indicates that Sw-CNc 1000 has a more ordered graphitic structure with fewer defects, which is critical for improving the electrochemical performance and stability of the material.

The XRD patterns presented in Figure 5c,d confirm the successful conversion of CNcs into a porous nanocarbon material following the annealing process. A broad peak, characteristic of the (002) plane of graphite, is observed between 20° and 26°, indicating the formation of graphitic carbon [41,42]. However, the XRD signal is weak and exhibits significant broadening, owing to the small particle size and limited crystallite dimensions. This broadening is attributed to the Scherrer effect, whereby a smaller crystallite size leads to increased peak width, indicating a limited long-range order within the carbon structure. In addition, the presence of broadened peaks reflects a higher degree of structural disorder and defects in the graphitic lattice, which are typical for nanostructured materials. In such materials, the reduced coherence length of the lattice and the presence of imperfections lead to a less-defined crystallographic structure. The weak and broad peaks suggest that while the CNcs were converted to graphitic carbon, the crystallites were relatively small and had lower graphitization than bulk graphite, potentially due to the limited annealing temperature or the inherent properties of the carbon precursor. These results demonstrate that the CNcs were successfully transformed into graphitic carbon, albeit with a limited degree of crystallinity, which is expected for nanostructured carbon materials [43,44,45].

XPS was used to analyze the surface chemistry and electronic states of the carbon materials. This technique provides detailed information on the presence of specific chemical groups, including oxygen-containing functional groups, such as C-O, –C=O, and–COO, which are critical to the surface reactivity of the material. Additionally, XPS allows for the precise detection of structural defects, such as vacancies, grain boundaries, and disordered regions, which affect the overall properties of the material. By presenting binding energy shifts and peak intensities according to material composition, XPS offers a comprehensive understanding of the chemical composition and electronic structure of a material, providing insights into the extent of graphitization, defect density, and impact on the electrochemical and conductive properties [46,47].

**Figure 6:**
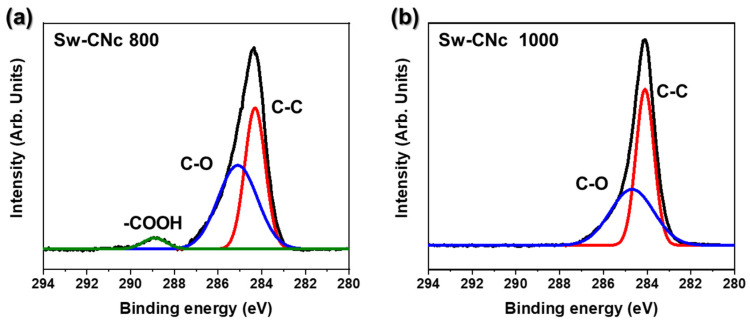
XPS profiles of (**a**) SW-CNc 800 and (**b**) SW-CNc 1000.

Figure 6 shows the appearance of an XPS C1s peak centered at 284.4 eV, corresponding to carbon, after the annealing of the CNc samples at 800 and 1000 °C. Sw-CNc 800 (Figure 6a) exhibits a strong sharp peak at 284.4 eV (C-C), indicating high-quality graphite, and weaker peaks at 285.4 (C-O) and 286.8 eV (-COO), which are derived from the CNc in seaweed waste. In comparison, the -COO peak disappeared, the intensity of the C-O peak decreased, and the C-C peak became more pronounced for Sw-CNc 1000, as shown in Figure 6b. These changes suggest that the degree of carbonization improved as the annealing temperature increased, leading to a reduction in the number of oxygen-containing functional groups. The decrease in the oxygen functional groups and increase in the C-C bonds suggest improved structural ordering and graphitization, indicating the formation of a more crystalline and conductive carbon network at elevated temperatures.

**Figure 7:**
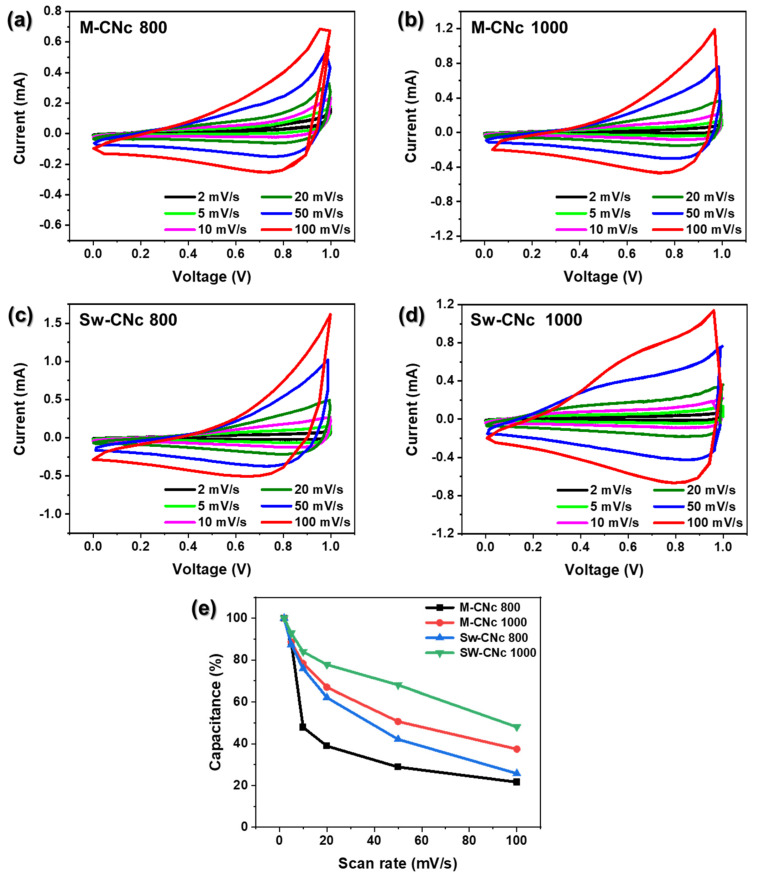
Electrochemical characterization of the electrodes based on porous nanocarbon materials derived from M-CNc and Sw-CNc. CV curves for (**a**) M-CNc 800, (**b**) M-CNc 1000, (**c**) Sw-CNc 800, and (**d**) Sw-CNc 1000. (**e**) Variation in the specific capacitance of the electrodes as a function of the scan rate.

Figure 7a–d show the CV characteristics of the EDLCs using the porous nanocarbon material over a wide scan rate range of 2–100 mV/s. At a low scan rate, the CV curves exhibit a relatively quasi-rectangular shape. This is because the porous nanocarbon materials have pores of various sizes that facilitate the reversible adsorption–desorption reactions of the electrolyte and ions [48]. However, the peaks became irregularly shaped at high scan rates. This deviation from the typical rectangular shape of the CV curves suggests the presence of pseudocapacitance, likely due to the redox-active functional groups, such as hydroxyl, carbonyl, and carboxyl groups, on the surface of the porous nanocarbon. These Faradaic reactions, which involve charge transfer across the electrode–electrolyte interface, enhance the capacitance of the electrode. The resulting non-rectangular CV profile reflects the hybrid charge storage mechanism, combining double-layer capacitance and pseudocapacitance, of the porous nanocarbon-based electrodes [49,50]. Figure 7e shows the variation in capacity according to the scan rate. Supercapacitors using porous nanocarbon materials as electrodes experience difficulties in the diffusion of electrolyte ions into the electrode structure, particularly their pores, as the scan rate increases, resulting in inefficient interactions between the electrolyte and porous nanocarbon material, and the non-electrolytic capacity decreases. The porous nanocarbon derived from Sw-CNc 1000 exhibited the optimal capacity and stability. This observation suggests that a significant number of mesopores were generated under the specific preparation conditions, providing an efficient and continuous ion transport pathway. Consequently, the accessibility of the electrolyte to the microporous regions was significantly enhanced, which contributes to the overall electrochemical performance of the material.

## 3. Materials and Methods

### 3.1. Materials

Seaweed waste was collected during summer from the Wando Province, Republic of Korea. Microcrystalline cellulose (MC, 20–100 μm) was supplied by Daejung, sulfuric acid (H_2_SO_4_, 95.0%) by Samchun, hydrogen peroxide (H_2_O_2_, 30%) by Junsei, ethanol (EtOH, 95%) by Ducksan, and tert-butanol (≥99.5%) by Sigma Aldrich. All chemicals were used as received without any additional purification.

### 3.2. Methods

#### 3.2.1. Isolation of CNcs from Seaweed Waste

The seaweed waste was first washed with distilled water to remove impurities and then completely dried in a vacuum oven. Once dried, it was ground into small particles, stirred in EtOH, and maintained at 80 °C for 1.5 h. The swollen seaweed particles were pre-irradiated with an E-beam (2.5 MeV and 1.5 mA) at 20, 40, and 60 kGy. The mixtures were then washed multiple times with EtOH to remove contaminants. Subsequently, the E-beam-irradiated seaweed wastes were reacted with 0.5, 1.0, 3.0, and 6.0 M H_2_SO_4_ added to a small amount of H_2_O_2_, and heated at 45 °C for 6, 12, and 24 h. The resulting mixtures were centrifuged for 10 min at 4000 rpm and washed with distilled water until the pH reached 6–7. The slurry was then dispersed in tert-butanol. Subsequently, microcrystalline CNcs (M-CNcs) and seaweed waste-based CNcs (Sw-CNcs) were prepared.

#### 3.2.2. Fabrication of Porous Nanocarbon Materials

The thermal annealing of the CNc samples was conducted by heating them to 800 or 1000 °C at a heating rate of 10 °C/min under a mixed Ar/H_2_ atmosphere and holding them at the final temperature for 30 min. After being cooled to ambient temperature, the prepared porous nanocarbon materials were immersed in a mild etchant to remove contaminants, filtered, and washed multiple times with EtOH. Finally, free-standing porous nanocarbon materials were obtained by drying at 40 °C in a vacuum oven for a sufficient time. The samples were designated as M-CNc 800, M-CNc 1000, Sw-CNc 800, and Sw-CNc 1000 according to the CNc type and annealing temperature.

### 3.3. Characterization

Morphological characterization was conducted using scanning electron microscopy (SEM; S-4800 microscope, Hitachi Corp., Chiyoda City, Japan). The structural properties were characterized using dynamic light scattering (DLS, Zetasizer Nano-ZS ZEN 3600, Malvern Corp., Westborough, MA, USA), X-ray diffraction (XRD, MXD10, Rigaku Corp., Tokyo, Japan), laser scanning confocal micro-Raman spectrometry (AFM-Raman, Alpha300s, WITec Corp., Ulm, Germany), and X-ray photoelectron spectroscopy (XPS, K-alpha, Thermo Fisher, Waltham, MA, USA).

### 3.4. Electrochemical Characterization

Electrochemical measurements were performed using a WBCS 3000 battery tester (WonA-Tech, Korea) under ambient conditions. A three-electrode system consisting of a Pt mesh counter electrode, an Ag/AgCl reference electrode, and the porous nanocarbon material deposited on stainless steel as the working electrode was employed. All the tests were performed in a saturated 1 M H_2_SO_4_ electrolyte. The working electrode was fabricated by mixing 80 wt% porous nanocarbon material, 10 wt% carbon black, and 10 wt% polyvinylidenedifluoride in N-methyl pyrrolidinone. After being homogenized, the resulting paste was coated onto pre-cleaned stainless steel substrates and dried overnight at 120 °C in a vacuum oven. Cyclic voltammetry (CV) was performed at scan rates ranging from 2 to 100 mV/s. The specific capacitance was calculated as follows:

$$C=\frac{1}{m\cdot ∆V\cdot v}\int IdV$$

where *C* is the specific capacitance (F/g), *I* is the current (A), ∆*V* is the applied potential window (V), and *m* is the total mass of the active material (g).

## 4. Conclusions

This study successfully demonstrates the isolation of CNcs from seaweed waste and their conversion into a porous nanocarbon material through a refined process involving E-beam irradiation, acid hydrolysis, and thermal annealing. The utilization of seaweed, which is naturally low in lignin and hemicellulose, as a biomass source offers an environmentally friendly and efficient approach for CNc isolation compared with that used for traditional wood biomass. The application of E-beam irradiation proved pivotal in enhancing the efficiency of CNc isolation by significantly reducing the particle size, shortening the reaction time, and minimizing the use of chemical reagents.

The porous nanocarbon materials derived from the seaweed waste-based CNcs (i.e., Sw-CNc) displayed remarkable electrochemical performance when employed as electrode materials in EDLCs. Specifically, the Sw-CNcs 1000 exhibited an excellent capacity and stability, largely owing to the formation of a dense network of mesopores that enabled smooth ion transport and enhanced electrolyte accessibility. Furthermore, the superior crystallinity of carbon and reduced structural defects, as confirmed by Raman spectroscopy, XPS, and XRD analyses, further contributed to the improved electrochemical performance of Sw-CNc 1000.

In conclusion, this study introduces a novel and sustainable method for converting seaweed waste into advanced porous nanocarbon materials, demonstrating significant potential as electrode components in energy storage devices, including active materials and conductive agents. This approach not only addresses the environmental issues related to seaweed waste but also provides a scalable and cost-effective pathway for the synthesis of high-performance carbon materials, offering broad applicability in energy storage technologies.

## Data Availability

The data presented in this study are available in this article.
